# Meaning-Centered Psychotherapy for Cancer Caregivers Study protocol of a randomized controlled trial

**DOI:** 10.21203/rs.3.rs-7963562/v1

**Published:** 2026-02-04

**Authors:** Allison J. J. Applebaum, Leah E. Walsh, Talia Zaider, Raymond E. Baser, Morgan J. Loschiavo, William S. Breitbart, Wendy G. Lichtenthal

**Affiliations:** Icahn School of Medicine at Mount Sinai; Icahn School of Medicine at Mount Sinai; Memorial Sloan Kettering Cancer Center; Memorial Sloan Kettering Cancer Center Department of Epidemiology & Biostatistics; Memorial Sloan Kettering Cancer Center; Memorial Sloan Kettering Cancer Center; University of Miami Hospitals and Clinics: Sylvester Comprehensive Cancer Center

**Keywords:** Cancer caregiver, caregiver support, Meaning-Centered Psychotherapy for Cancer Caregivers, meaning, purpose, spiritual wellbeing, existential distress, advanced cancer, advance care planning, bereavement

## Abstract

**Background:**

Caregivers of patients with advanced cancer shoulder immense responsibilities as they care for patients, including symptom and medication management, providing emotional support, and navigating healthcare treatment and decision-making. Due to the heavy toll of these responsibilities, caregivers are at high risk for profound mental health challenges, including anxiety, depression, and posttraumatic stress disorder. A key driver of this heightened risk for psychopathology in caregivers is existential distress, manifesting as a loss of meaning and purpose, decreased spiritual well-being, and hopelessness. Historically, psychosocial interventions targeting distress in cancer caregivers have neglected to address existential distress. Meaning-Centered Psychotherapy for Cancer Caregivers, a 7-session structured intervention, was developed to address this gap. In a pilot randomized controlled trial, the approach led to enhancements in personal meaning, benefit finding, and spiritual well-being. Here, we present a large, multi-site trial that aims to definitively examine the efficacy of Meaning-Centered Psychotherapy for Cancer Caregivers in an adequately powered study.

**Method:**

This randomized controlled trial will evaluate the efficacy of Meaning-Centered Psychotherapy for Cancer Caregivers versus Supportive Psychotherapy for Cancer Caregivers on primary (personal meaning and spiritual well-being) and secondary (anxiety, depression, sense of meaning in caregiving, benefit finding, caregiver burden, social support) outcomes at baseline, post-treatment, and at 6- and 12-months follow-up. It will also evaluate the role of sense of meaning in life as a mediator of secondary outcomes, as well as the impact of Meaning-Centered Psychotherapy for Cancer Caregivers on pre-and post-loss bereavement outcomes. Two hundred caregivers of patients with advanced (stage III/IV) solid tumor cancers from Memorial Sloan Kettering Cancer Center, Sylvester Comprehensive Cancer Center, and the community will be enrolled.

**Discussion:**

Meaning-Centered Psychotherapy for Cancer Caregivers has the potential to help alleviate existential suffering in caregivers as they manage the multifaceted demands of caring for patients with advanced cancer. This trial seeks to evaluate the efficacy of this intervention in a more robust and representative trial of cancer caregivers, and extends prior research to explore mediators of improvement and the impact of the intervention on pre- and post-loss bereavement outcomes.

**Trial registration:**

This trial is registered at ClinicalTrials.gov, NCT06307535, registered on 03/05/2024

## Administrative information

Note: the numbers in curly brackets in this protocol refer to SPIRIT checklist item numbers. The order of the items has been modified to group similar items (see http://www.equator-network.org/reportingguidelines/spirit-2013-statement-defining-standard-protocol-items-for-clinical-trials/).

**Table:** 

Title {1}	Meaning-Centered Psychotherapy for Cancer Caregivers (MCP-C): Study protocol of a randomized controlled trial
Trial registration {2a and 2b}.	ClinicalTrials.gov, NCT06307535, registered on 03/05/2024 https://clinicaltrials.gov/study/NCT06307535
Protocol version {3}	Icahn School of Medicine at Mount Sinai, July 22, 2025
Funding {4}	This study is supported by the National Cancer Institute (R01CA285621).
Author details {5a}	Allison J. Applebaum, PhD^1^
	Leah E. Walsh, PhD^1^
	Talia Zaider, PhD^2^
	Raymond E. Baser^3^
	Morgan Loschiavo^2^
	William S. Breitbart^2^
	Wendy G. Lichtenthal^4^
	^1^Brookdale Department of Geriatrics and Palliative Medicine, Icahn School of Medicine at Mount Sinai
	^2^Department of Psychiatry and Behavioral Sciences, Memorial Sloan Kettering Cancer Center
	^3^Department of Epidemiology and Biostatistics, Memorial Sloan Kettering Cancer Center
	^4^Sylvester Comprehensive Cancer Center, University of Miami Health System
Name and contact information for the trial sponsor {5b}	This study is funded by the National Institutes of Health (R01CA28561).
Role of sponsor {5c}	This study is funded by the National Institutes of Health (R01CA285621).

## Introduction

### Background and rationale {6a}

Over 63 million individuals in the United States are caregivers, with a significant portion providing care to patients with life-limiting cancers.^1^ Caregivers of patients with cancer take on tremendous responsibilities across the illness trajectory, assisting with symptom management,^2^ providing emotional support^3,4^ and navigating the healthcare team and treatment,^5^ all while managing other personal, family, occupational, and financial responsibilities. The burden of cancer caregiving can lead to increased levels of anxiety, depression,^6^ and posttraumatic stress.^7^ Without timely intervention, such distress has the potential to negatively impact the care provided to patients^8–10^ and can contribute to poor adjustment and psychosocial outcomes in bereavement,^11,12^ such as prolonged grief disorder.^13^

A key driver of these mental health outcomes is existential distress, characterized as a loss of meaning and purpose in life, decreased spiritual well-being, and hopelessness.^14,15^ Existential distress is common among caregivers due to the competing demands of caregiving and personal life goals, and balancing hope for the present with an awareness of potential patient death in the future. Witnessing prolonged or aggressive medical treatment, patient suffering, or a decline in patient functioning can also contribute to existential distress.^16–18^ Importantly, caregivers who experience existential distress are less likely to engage in advance care planning discussions with patients and healthcare professionals, which ultimately negatively impacts the care patients receive at the end of life.^19^ As such, addressing existential distress in cancer caregivers has profound benefits for caregivers, as well as the patients for whom they provide care. When existential distress is mitigated, caregivers are better able to cultivate deeper connections with patients and strengthen their sense of meaning in caregiving. This in turn can reduce regret and unfinished business and prevent poor bereavement outcomes.^20–22^ Despite these facts, existential distress has historically remained unaddressed in psychosocial interventions for cancer caregivers.

Meaning-Centered Psychotherapy (MCP)^23,24^ is an empirically supported intervention originally developed to help patients with advanced, life-limiting cancers connect to meaning and purpose, despite the challenges they face. Our team adapted MCP to assist cancer caregivers to connect to meaning and purpose and mitigate existential distress (Meaning Centered Psychotherapy for Cancer Caregivers; MCP-C).^25,26^ Developed with feedback from caregiver stakeholders, MCP-C is an existentially-oriented 7-session manualized intervention that helps caregivers connect – or reconnect – to sources of meaning and purpose in their life despite the challenges, limitations, and losses that come with caregiving. Our pilot trial of MCP-C among 60 caregivers of patients with glioblastoma evidenced strong preliminary effects on enhanced personal meaning, benefit finding, and spiritual well-being. The results of this pilot study, alongside qualitative feedback from participants that supported its feasibility and acceptability,^27^ suggested that MCP-C should be tested in a larger, fully powered randomized controlled trial.

### Objectives {7}

This randomized controlled trial has three objectives. First, this study will investigate the efficacy of MCP-C versus Supportive Psychotherapy for Cancer Caregivers (SP-C) on primary (i.e., sense of meaning in life, spiritual well-being) and secondary (i.e., anxiety, depression, sense of meaning in caregiving, benefit finding, caregiver burden, social support) outcomes immediately post treatment and explore maintenance in improvements up to 1-year post treatment (Aim 1). Second, this study will evaluate the role of sense of meaning in life as a mediator of improvements in key secondary outcomes (Aim 2). Third, this study will explore the effects of MCP-C on pre- and post-loss grief and bereavement outcomes (Aim 3).

### Trial design {8}

This is a randomized controlled trial in which participating caregivers (N = 200) are randomized 1:1 to either MCP-C or SP-C to test the efficacy of MCP-C versus SP-C.

## Methods: Participants, interventions and outcomes

### Study setting {9}

The coordinating site for this trial is the Icahn School of Medicine at Mount Sinai (ISMMS). Participants are being recruited from two primary sites: Memorial Sloan Kettering Cancer Center (MSK) and Sylvester Comprehensive Cancer Center (SCCC), part of the University of Miami Health System. Caregivers will also be recruited through the community via the National Alliance for Caregiving and the American Cancer Society, who will share information about this trial with their listservs and on their social media platforms. Information about study participation will be included on all advertisements and posts and caregivers will be directed to contact ISMMS to learn more about study participation.

### Eligibility criteria {10}

Participants are eligible if they are: at least 18 years of age, can read and understand English, a self-reported current caregiver to a patient with Stage III or IV solid tumor cancer currently receiving any type of medical treatment (e.g., curative, palliative), reporting caregiving-related distress as evidenced by scoring a 4 or greater on an adapted Distress Thermometer (DT) for caregivers, and residing in New York, New Jersey, Florida, or Connecticut, or have the ability to complete sessions in compliance with current telehealth regulations. Participants are not eligible if they do not have a reasonable understanding of study procedures as judged by the consenting professional or if they are engaged in regular individual psychotherapeutic support that they are unable or unwilling to pause while receiving the study intervention.

### Who will take informed consent? {26a}

Study team members trained in informed consent procedures will screen eligible participants, explain study procedures, and enroll caregivers who meet study inclusion criteria and provide informed consent.

### Additional consent provisions for collection and use of participant data and biological specimens {26b}

N/A

## Interventions

### Explanation for the choice of comparators {6b}

We are comparing MCP-C, the study intervention, to SP-C. SP-C is based on a model of Supportive Psychotherapy that has been used in previous randomized trials of MCP as the comparator intervention and was adapted to represent the standard of caregiver support in the community. It has also been standardized for use in RCTs.^28^

### Intervention description {11a}

#### Meaning-Centered Psychotherapy for Cancer Caregivers (MCP-C).

MCP-C is a 7-session intervention delivered individually over telehealth platforms that combines didactics, discussion, and experiential exercises focused on themes related to meaning and caregiving. Session 1 (*Concepts and Sources of Meaning*) provides an overview of meaning and caregiving and asks participants to share their caregiving journey and their understanding of the word “caregiver.” Session 2 (*Identity Before and After Becoming a Caregiver*) focuses on how caregiving impacts participants’ identities, highlighting both immutable aspects as well as the possibility for growth. Session 3 (*Historical Sources of Meaning*) focuses on the meaning derived through reflecting on one’s past legacy (i.e., family history and past life experiences that one does not choose), the present legacy caregivers are actively creating, and the future legacy they will give to others. Session 4 (*Attitudinal Sources of Meaning*) focuses on helping caregivers to recognize where they have choice and how meaning can be derived through reflecting on how they choose to respond to life’s limitations and challenges, including how they choose to face the potential eventual death of their care partner. Session 5 (*Creative Sources of Meaning*) focuses on meaning generated through creating one’s life (i.e., the activities they do or pursuits or interests that make them who they are), and highlights self-care as a key route through which caregivers can continue to create their lives, despite the challenges of caregiving. Session 6 (*Experiential Sources of Meaning*) focuses on meaning derived through experiencing life through the five senses and through the experiences of love, beauty, and humor. Session 7 (*Transitions*) reviews the material covered and provides space to reflect on the ways participants can continue to use these sources of meaning as resources moving forward. At the end each session, caregivers are encouraged to reflect on questions specific to the subsequent session’s theme for homework to help guide discussion and reflection.

#### Supportive Psychotherapy for Cancer Caregivers (SP-C).

SP-C is a 7-session intervention delivered individually over telehealth platforms based on conceptual models developed by Rogers^29^ and Block.^30^ SP-C incorporates client-centered, supportive strategies and offers caregivers a safe space to share challenges they face in their daily routines and experiences. For each session, the topic of discussion is determined by the caregiver and centers on their most pressing caregiving-related need at that time. Unlike in MCP-C, the therapist does not impose a structure or agenda onto the session. The role of the therapist is to provide supportive reflections, to acknowledge and validate their emotions and reactions in response to the caregiving role, to encourage caregivers’ self-reflection, and to highlight adaptive coping responses to challenges, including support-seeking and self-care strategies. Therapists utilize curiosity, acceptance, and openness to facilitate the therapeutic alliance to assist caregivers to explore and validate their experiences. SP-C has been utilized and standardized in past RCTs of MCP and tailored for cancer caregivers.

### Criteria for discontinuing or modifying allocated interventions {11b}

Discontinuing the allocated intervention will only occur if the participant chooses to end their participation in the study or are no longer able to comply with telehealth regulations. Participants will not be able to modify their allocated intervention arm once randomized. There are no significant concerns of harm from either study arm that would require a change in study arm allocation. If participants become bereaved, they are welcome – and encouraged – to remain on the study, and bereavement-related adaptations are made to subsequent sessions.

### Strategies to improve adherence to interventions {11c}

We encourage participants to complete study sessions weekly and will be flexible with their schedule if they need to reschedule a session for a later date. We expect that the 7 study sessions will be completed within 14-weeks. This schedule flexibility will help to improve adherence to the intervention. All intervention sessions are audio/video recorded and a random sample of 30% of cases (100% of sessions for these participants) will be evaluated and rated for treatment integrity by independent raters throughout the study. We have developed comprehensive treatment manuals for MCP-C and SP-C to facilitate standardized delivery, and all interventionists undergo a half day training in either MCP-C or SP-C, followed by weekly group supervision sessions. Supervisors also review recorded sessions to facilitate reinforcement of model-specific strategies and alert therapists of any drift in model adherence. To reduce likelihood of treatment contamination, each condition is delivered by distinct treatment providers. Further, therapists monitor attendance and homework completion when relevant as part of session notes. Deviations from the protocol are recorded and discussed regularly during weekly group supervision.

### Relevant concomitant care permitted or prohibited during the trial {11d}

Caregivers will be ineligible for participation if they are engaged in regular individual psychotherapeutic support that they are unable or unwilling to put on hold for the course of the intervention. The use of psychotropic medications while engaging in the trial is permitted.

### Provisions for post-trial care {30}

There are no provisions for post-trial care. However, should a participant specifically request referrals for ongoing support after completion of the trial, the study team will provide appropriate resources.

### Outcomes {12}

The primary outcome measures are sense of meaning in life and spiritual well-being. Secondary outcome measures include anxiety and depression, sense of meaning and purpose in caregiving, benefit finding, caregiver burden, and social support. Pre-loss (preparedness for patient death, pre-loss grief intensity, risk for bereavement mental health challenges, engagement in advance care planning) and post-loss (presence of regret, unfinished business, post-loss grief intensity, risk for post-loss bereavement mental health challenges, and occurrence of advance care planning discussions and goal-concordant care at patients’ end of life) bereavement outcomes will also be explored. The SPIRIT figure for the trial is shown in Fig. 2.

### Participant timeline {13}

**Figure 1:**
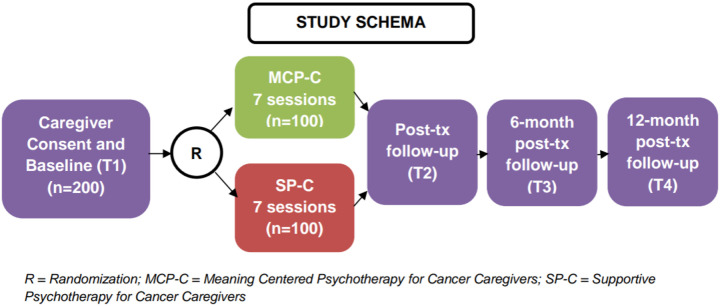
Study timeline.

**Figure 2:**
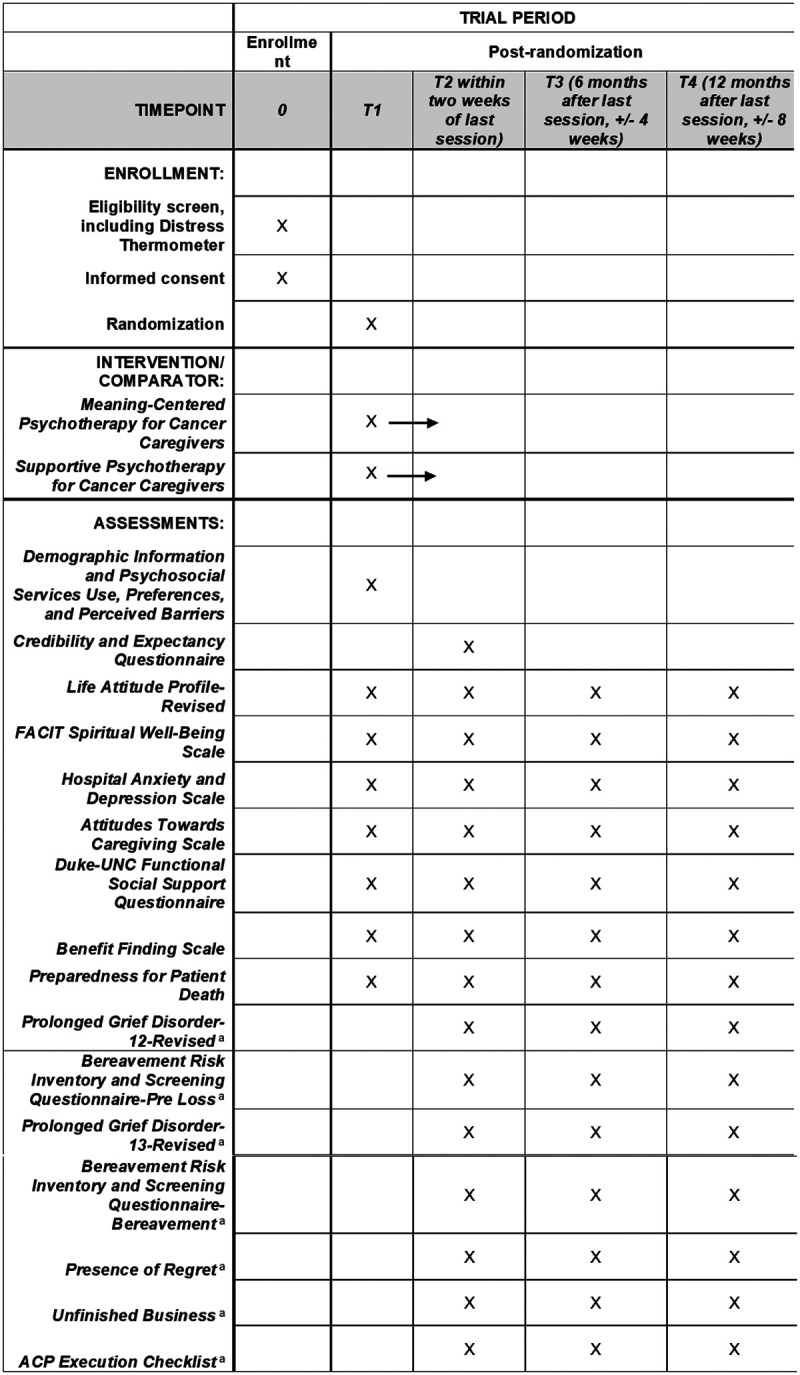
SPIRIT Figure Participant timeline: Schedule of enrollment, interventions, and assessments. ^a^This measure is only completed for the timepoints after which a participant has become bereaved, if applicable. These questionnaires are not completed by participants who are not bereaved.

Potential participants will be screened for eligibility (approximately 5 minutes) and will meet criteria if they report distress > 4 on the Distress Thermometer and indication that the distress is related to caregiving. If interested, participants will complete informed consent procedures and will be offered to immediately complete baseline (T1) assessments. Participants will have up to 45 days (consent expiration) to complete baseline assessments. Once baseline assessments are complete, they will be randomized to either MCP-C or SP-C and assigned to a therapist for 7 individual sessions that will be completed over Webex. Participants will have up to 14 weeks to complete all 7 sessions. After intervention completion, participants will complete follow-up assessments immediately post-treatment (T2, + 2 weeks), 6-month follow-up (T3, +/− 4 weeks) and 12-month follow-up (T4, +/− 8 weeks). See Fig. 1 for the full study timeline.

### Sample size {14}

Participants will be 200 caregivers recruited across study sites and the community, with 100 caregivers randomized to each study arm (MCP-C vs. SP-C). The sample size was chosen to provide 80% power to detect reasonable treatment effect sizes for our two primary endpoints while maintaining the Type 1 error rate at α = 0.05 for these two comparisons. Using methods relevant to clinical trials with repeated measures,^31^ we calculated the smallest effect size that we will be able to detect for the immediate post-treatment coefficient of the time-by-arm interaction term in our linear mixed model analysis of one of the two primary outcome measures (LAP-R PMI). Given the sample size (*N* = 200), we will be able to detect a standardized mean difference treatment effect size between the two study arms of 0.48 standard deviations. This calculation assumed a correlation between baseline and follow-up measures of 0.5, 20% attrition by the post-treatment assessment, a two-sided significance test, and a type I error of .025 (0.05 overall for both primary endpoints).

### Recruitment {15}

To maximize recruitment and retention, this study employs a multi-site design where participants will be recruited from outpatient clinics in various departments at MSK and SCCC. At both sites, we will employ three recruitment strategies. Flyers will be posted (digitally or using paper with the clinic’s permission) in clinics at MSK and SCCC. Study contact information will be provided on each flyer where caregivers can reach out to learn more about the study. Participants will also be recruited through patient appointments. Physicians will either directly provide caregivers with the contact information of the study team, or the study team will approach caregivers during or after patients’ next clinic appointment and introduce the study. Third, if clinicians who are aware of the study receive an inquiry from a caregiver about supportive services, they will provide their information to the study team who will subsequently send a study invitation letter to the caregiver via email. The study team will then make 3–5 follow-up calls/emails to discuss interest and participation with the caregiver.

Recruitment from community partners will occur through listservs and social media channels (e.g., Twitter/X, Facebook) of both the American Cancer Society and the National Alliance for Caregiving in states where MCP-C and SP-C supervisors are licensed (New York, New Jersey, Florida, and Connecticut). These advertisements will include study team information and will direct potential participants to contact the study team by email. Caregivers from New York and New Jersey will be enrolled at MSK, and those from Florida and Connecticut will be enrolled at SCCC. During the initial approach and informed consent conversation, study team members will ensure that participants reside in a state where the MCP-C and SP-C supervisors are licensed and may be asked to provide certain health information that is necessary for the recruitment and enrollment process.

## Assignment of interventions: allocation

### Sequence generation {16a}

Participants will be randomized to either MCP-C or SP-C using MSK’s Clinical Research Database, a secure computer system that ensure allocation concealment. Randomization will be 1:1 (MCP-C:SP-C) stratified by study site (MSK, SCCC, Community/MSK, and Community/SCCC) using randomly permuted blocks of random length. Given the clear focus of MCP-C versus SP-C on unique meaning-focused themes, participants and their study interventionists will not be blinded to intervention assignment. The study biostatistician will also not be blinded to treatment assignment.

### Concealment mechanism {16b}

N/A.

### Implementation {16c}

Participants will be randomized 1:1, stratified by study site, to MCP-C or SP-C using MSK’s Clinical Research Database, a secure computer system that ensures allocation concealment that uses randomly permuted blocks of random length to allocate participants to study arm. Participants will be enrolled by a consenting professional at the corresponding study site.

## Assignment of interventions: Blinding

### Who will be blinded {17a}

Neither participants nor their study interventionists will be blinded to intervention assignment due to the clear focus of the MCP-C intervention on meaning and existential content and SP-C on the provision of support.

### Procedure for unblinding if needed {17b}

N/A.

## Data collection and management

### Plans for assessment and collection of outcomes {18a}

Data will be collected via self-report measures using the Research Electronic Data Capture (REDCap), a secure web application for creating and managing online survey databases. The Clinical Research Coordinator (CRC) will send all study assessments to participants at the appropriate timepoint. Outcomes will be measured at four timepoints: baseline, directly after informed consent or until consent expiration (i.e., 45 days; T1), immediately post-intervention + 2 weeks (T2), 6-month follow-up +/− 4 weeks (T3), and 12-month follow-up +/− 8 weeks (T4). This will allow flexibility for study assessment completion and aid caregiver retention throughout the entire study. All data collection forms can be found in the study protocol.

At baseline, (T1), participants will complete demographic information (e.g., age, sex, race, ethnicity, income), caregiving characteristics (e.g., relationship to the patient, length of caregiving), and a survey about psychosocial service use, preferences, and perceived barriers, using established questionnaires from our group’s previous studies. Participants will also complete all outcome measures (i.e., sense of meaning in life, spiritual well-being, anxiety and depression, benefit finding, meaning in caregiving, caregiver burden, and social support) at T1. At T2, in addition to study outcome measures, participants will complete the Credibility and Expectancy Questionnaire (CEQ) to assess treatment acceptability and perceived effectiveness. Pre-loss (preparedness for patient death, pre-loss grief intensity, risk for bereavement mental health challenges, engagement in advance care planning) and post-loss (presence of regret, unfinished business, post-loss grief intensity, risk for post-loss bereavement mental health challenges, and occurrence of advance care planning discussions and goal-concordant care at patients’ end of life) bereavement outcomes will also be explored at all time points.

## Description of study measures

### Screening and non-outcome measures

#### Caregiving-related distress.

A modified version of the Distress Thermometer^32^ will be used to screen for distress related to caregiving. Participants are asked to rate their distress on a scale of 0–10 (cutoff score > 4) and indicate that their distress is related to caregiving to be eligible for the study. The Distress Thermometer will be administered at screening only.

#### Treatment Acceptability and Perceived Effectiveness.

The CEQ is a 6-item measure of treatment acceptability and perceived effectiveness.^33,34^ This measure has been used extensively in prior research and has strong psychometric properties.^34^ The CEQ is administered at T2 only.

### Primary outcomes

#### Sense of Meaning in Life.

The LAP-R PMI^35^ is a 48-item measure of meaning in life, with items rated on a 7-point Likert scale from *Strongly Agree* to *Strongly Disagree*, with higher scores indicating a stronger presence of that type of meaning. The LAP-R generates two composite scores: the Personal Meaning Index (PMI) and Existential Transcendence (ET). The LAP-R has demonstrated strong internal consistency in previous research (Cronbach’s α range: .77–.91).^35^ The LAP-R PMI is administered at T1-T4.

#### Spiritual Wellbeing.

The FACIT Spiritual Well-Being Scale (SWBS)^36^ is a 12-item measure of spiritual well-being measured on a 0 (Not at all) to 4 (very much) scale, with higher scores indicating higher spiritual wellbeing. This measure has demonstrated strong internal consistency across all subscales in cancer caregivers (Cronbach’s α range: .88–.93).^26^ The SWBS is administered at T1-T4.

### Secondary outcomes

#### Anxiety and Depressive Symptoms.

The HADS is a 14-item measure of anxiety and depressive symptoms with items rated on a 0 to 3 scale, with higher scores indicating greater symptomatology. A total score and anxiety and depression subscale scores are generated. The total HADS and its subscales has demonstrated strong internal consistency in cancer caregiver populations (Cronbach’s α range: .82–.92).^26,37^ The HADS is administered at T1-T4.

#### Sense of Meaning and Purpose in Caregiving.

The Attitudes Toward Caregiving Scale (ATCS)^38^ is a 43-item measure rated on a 5-point Likert-type scale that examines the extent to which caregivers find meaning in caregiving. Scores can be summed for a total score in addition to three subscales: loss/powerlessness, provisional meaning, and ultimate meaning, with higher scores indicating higher overall meaning and purpose. The ATCS is administered at T1-T4, in non-bereaved caregivers.

#### Benefit Finding.

The BFS is a 17-item measure of perceived benefit finding that has been modified for careger populations. It has been used in our group’s previous research and has demonstrated strong internal consistency (Cronbach’s α range: .91 to .95.^39,40^ The BFS is administered at T1-T4.

#### Caregiver Burden.

The CRA is a 24-item measure with items scored on a 5-point Likert-type scale.^41^ Items are summed for a total score (range: 24–120) across five subscales: self-esteem, family support, impact on finances, schedule, and health. The CRA is a widely used measure of caregiver burden that has demonstrated good internal consistency and construct validity.^42^ The CRA is administered at T1-T4 in non-bereaved caregivers.

#### Social Support.

The FSSQ is an 8-item measure of perceived social support. Items are summed to a total score ranging from 0 to 40, with higher scores indicating greater perceived social support. The FSSQ has demonstrated strong internal consistency (Cronbach’s α = .94).^43^ The FSSQ will be administered at T1-T4.

## Bereavement related outcomes

### Pre-Loss Outcomes:

#### Preparedness for Patient Death.

The Preparedness for Patient Death item asks participants “If your care partner were to die soon, how prepared would you be for his/her death?” Response options are “not at all,” “somewhat,” and “very.” This item is based on previous studies of preparedness for loss.^44–46^ Preparedness for patient death is measured at T1-T4 in non-bereaved caregivers.

#### Pre-loss grief intensity.

The Prolonged Grief-12-Revised (PG-12-R) is a 12-item measure of pre-loss grief, adapted from a measure of prolonged grief symptoms^44^ that is worded to reflect the patient as living. PG-12-R items are rated on a 5-point Likert-type scale, with total scores ranging from 11–55, with higher scores suggesting greater anticipatory grief. The PG-12-R has demonstrated strong internal consistency,^47^ and is administered at T1-T4 to non-bereaved caregivers.

#### Risk for Bereavement-Related Mental Health Challenges.

The Bereavement Risk Inventory and Screening Questionnaire-Pre-Loss (BRISQ-P) is a 30-item screener for bereavement mental health challenges, with items rated on a 5-point Likert scale.^48^ The BRISQ-P assesses illness or death-related and bereavement-related risk factors and is administered at T1-T4 to non-bereaved caregivers.

#### Advance Care Planning Engagement.

Engagement in advance care planning will be measured by the Advance Care Planning (ACP) Checklist–Current Caregiver^49^ which includes 14 items that ask caregivers about previous ACP discussions and domains of ACP (e.g., prognosis, goals of care, execution of advance directives). Items are scored and summed to a total score. This checklist is administered at T1-T4 to non-bereaved caregivers.

### Post-Loss Outcomes:

#### Post-loss grief intensity.

The Prolonged Grief-13-Revised (PG-13-R) is a 13-item measure that evaluates symptoms of prolonged grief disorder.^50^ It assesses 10 grief-related symptoms on a 5-point Likert scale. Scores range from 10–50 with higher scores indicating greater grief intensity. The PG-13-R is administered at T2-T4 to caregivers who become bereaved during the study.

#### Risk for bereavement-related mental health challenges post-death.

The Bereavement Risk Inventory and Screening Questionnaire-Post-Loss (BRISQ-B) is a measure of risk for bereavement mental health challenges following the patient’s death.^48^ It is nearly identical to the BRISQ-P but items are modified to refer to the patient’s death rather than their illness. It also includes items related to the bereavement experience. Items are rated on a 5-point Likert-type scale. The BRISQ-B is administered at T2-T4 to caregivers who become bereaved during the study period.

#### Presence of Regret.

The single Presence of Regret item^22^ asks caregivers, “Do you regret any of your actions or choices that occurred while providing care to your loved one with cancer when they were ill?” with a dichotomous yes/no response option. For those who report yes, they will be asked to provide an example of their most troubling regret and to rate their distress about the stated regret on a 10-point scale (10 = most distressed). The Presence of Regret item will be administered at T2-T4 for those caregivers who become bereaved during the study period.

#### Unfinished business.

The single Unfinished Business item asks caregivers, “Do you feel that anything was unfinished, unsaid, or unresolved in your relationship with your loved one with cancer?” with a yes/no response option. If yes, caregivers are asked to provide an example of their most troubling source of unfinished business and to rate their distress about this on a 10-point scale (10 = most distressed). This item was developed to be face valid and consistent with theory on regret and unfinished business,^20,20,51^ and has demonstrated good concurrent validity.^21^

#### Advance Care Planning Engagement.

Caregivers who become bereaved will complete the Advance Care Planning Checklist–Past Caregiver, which mirrors the Advance Care Planning Checklist-Current Caregiver, but asks about what type of care the patient received at end of life, where the patient died, and if the location of death and care provided were consistent with the patient’s stated wishes.

### Plans to promote participant retention and complete follow-up {18b}

Participants will be compensated $10 for each intervention session they complete. They will also be compensated $5 for completing baseline assessments (T1), $10 for completing post-intervention assessments (T2), $15 for completing 6-month follow-up assessments (T3), and $20 for completing 12-month follow-up assessments (T4). Participants are sent a maximum of three emails and called a maximum of three times to remind them to complete each survey. Those participants who discontinue their involvement in the study intervention will be asked to continue to complete study assessments and will receive reimbursement for completed assessments.

### Data management {19}

A Clinical Research Coordinator (CRC) is assigned to this study whose responsibilities include project management and compliance, data collection, data entry, data reporting, regulatory monitoring, identifying and resolving problems, and coordinating activities related to the study protocol. The maintained dataset will include minimal data (race, ethnicity, sex, and age) and will be entered into the Clinical Research Administration Clinical Trials Management System.

Data collected for this study will be entered into and managed via the secure Research Electronic Data Capture (REDCap) Database. REDCap is an open-source platform that allows for data collection in a secure manner over a web-based interface. The REDCap platform was developed by Vanderbilt University with whom ISMMS has a standing agreement to allow the usage of REDCap for academic/research purposes.

To ensure data quality, reports will be generated periodically to monitor accrual and completeness of participant registration data. Data quality reports will also be generated to assess for missing data and inconsistencies. Throughout the study, data on accrual rates and follow-up will be monitored to identify any potential problems which will be brought to the attention of the study team during weekly team meetings. An audit of consent and eligibility data will be performed with the first five enrolled participants to assess for data completeness and data accuracy.

### Confidentiality {27}

Research staff involved in participant recruitment will provide an overview of the study, invite potential participants to participate, and discuss potential risks and benefits during the informed consent process. All participants are required to provide informed consent prior to completing study assessments and the intervention. Participants can choose to skip any question in the assessment battery they do not wish to answer. Participants who report experiencing psychological distress related to completing study questions will be offered support from the enrolling institutions’ counseling or supportive care services or receive community referrals for mental health providers.

When presenting study results, data will be presented for the entire sample and will not reference any individual participant’s data. All survey data will be de-identified using a code number to ensure confidentiality of participant data. For research staff who need to access study data, password security measures and other relevant restrictions will be applied to maintain confidentiality. Confidential data will only be accessible on a “need to know” basis for any particular research staff member. All personnel involved with the study will receive training in HIPAA and human subjects’ protections, and will receive information on the ethics of accessing electronic data.

### Plans for collection, laboratory evaluation and storage of biological specimens for genetic or molecular analysis in this trial/future use {33}

No biospecimen data will be collected as part of study aims and procedures.

## Statistical methods

### Statistical methods for primary and secondary outcomes {20a}

Descriptive statistics will be calculated for baseline demographic and caregiver clinical variables, summarizing each outcome by treatment arm. We will assess the distribution of each study outcome and, if necessary, perform corrective transformations before calculating inferential statistics. We will also perform all efficacy analyses by the intention-to-treat principle, meaning that all randomized participations will be analyzed regardless of drop-out or treatment adherence in their respective study arm. All endpoint comparisons between study arms will be adjusted for the randomization stratification variable (study site).

Aim 1 Analytic Plan: The primary and secondary outcome measures for Aim 1 are all continuous variables assessed repeatedly over the four timepoints: baseline (T1), immediately post-treatment (T2), 6-month follow-up (T3) and 12-month follow-up (T4). The co-primary efficacy endpoints for Aim 1 are the LAP-R PMI and FACIT SWBS at the immediately post-treatment assessment (T2). We will consider MCP-C efficacious if MCP-C participants have significantly greater improvements than SP-C participants in LAP-R PMI or FACIT SWBS scores at T2 at the p < .025 statistical significance threshold in the analysis described below. We will analyze each continuous primary and secondary outcome measure by using constrained linear mixed model (cLMM) regression.^52–54^ This statistical method accounts for within-subject correlations due to repeated measurement of participants and, under the reasonable missing at random (MAR) assumption, allows unbiased estimation of between-group differences without excluding participants with missing follow-up data. Each of these outcome measures will be analyzed using a separate cLMM regression with random intercept that will model the baseline and repeated follow-up scores (dependent variable) as a function of time (categorical) and the time-by arm interaction, controlling for the randomization stratification variable (study site). The cLMM method will also constrain the two study arms to have a common baseline mean on the outcome measure to reflect the pre-randomization nature of the baseline assessment (T1).^52^ All randomized participants will have, at a minimum, the baseline (T1) primary outcome assessments and will be included in the cLMM per the intention-to-treat principal. In the cLMM parameterization, the coefficients for the time-by-arm interaction are equivalent to the differences between arms in outcome measure score changes from baseline to the post-baseline time points, and they will be calculated with 95% confidence intervals (CIs). For each co-primary endpoint (LAP-R PMI and FACIT SWBS), we will conclude that MCP-C participants had significantly larger improvements in the endpoint at T2 compared to SP-C if the time-by-arm interaction coefficient for T2 is significantly larger than 0 at the *p* < .025 level. The secondary outcome measures will be compared between study arms using similar cLMM analyses. However, we will assess estimated treatment effects at each timepoint using 95% CIs instead of p-values.

Aim 2 Analytic Plan: We will use model-based causal mediation analysis (CMA)^55^ to evaluate whether changes in sense of meaning in life (LAP-R PMI) immediately post-treatment (T2) mediate the effect of treatment arm on benefit finding (BFS), anxiety (HADS-A), and depression (HADS-D) at 6-month follow-up. We will use the *mediation*^56^ package in R^57^ to conduct mediation analyses for each of the three outcomes (BFS, HADS-A, HADS-D). Each mediation analysis will estimate the average causal mediation effect (ACME, the effect of the treatment arm on the outcome via the mediator), average direct effect (ADE, effect of treatment arm on the outcome measure excluding the mediation), the average total effect (ATE, sum of the ACME and ATE), and the proportion of the ATE mediated by the mediator variable (ACME/ATE). To estimate these effects, we will input two models, (1) a mediator model and (2) an outcome model, to the estimation functions in the *mediation* R package. The (1) mediator model will be a linear regression with T2 LAP-R PMI as the dependent variable, and treatment arm and baseline (T1) LAP-R PMI as independent variables, controlling for baseline outcome measure (e.g., T1 BFS) and study site (randomization stratification variable). The (2) outcome model will be a linear regression with the T3 outcome measure (e.g., BFS) as the dependent variable, and treatment arm, baseline outcome measure, T2 and T1 LAP-R PMI, and study site (randomization stratification variable) as independent variables. We will conduct these mediation analyses once excluding those participants with missing data for any variables in the model, and we will conduct sensitivity analyses replicating the model after applying multiple imputation of missing data using chained equation analyses.^58^ Other sensitivity analyses will be conducted to evaluate results testing the impact of unmeasured pre-treatment confounding variables.^59^

Aim 3 Analytic Plan: Based on our group’s prior research, we estimate approximately 50 (25%) participants in this study will be become bereaved by the 12-month follow-up assessment (T4). Among the subset of non-bereaved caregivers, we will utilize the same cLMM analysis used in assessing the secondary outcomes in Aim 1 to examine differences between study arms in risk for bereavement mental health challenges (BRISQ-P) and pre-loss grief (PG-12-R). Multinomial logistic regression analyses will assess between-arm differences in T4 preparedness for loss in this subset, controlling for study site and baseline preparedness for loss. Among the subset of caregivers who become bereaved by T4, we will assess between-arm differences in T4 risk for bereavement mental health challenges (BRISQ-B), post-loss grief (PG-13-R), and presence of regret (single item, yes/no) using linear (BRISQ-B, PG-13-R) and logistic (presence of regret) regression models controlling for study site and time since bereavement. Also, among those bereaved before T4, we will test whether higher post-treatment (T2) preparedness for patient death and/or engagement in ACP discussions (ACP Execution Checklist score) are associated with lower levels of T4 post-loss grief (PG-13-R) by fitting linear regression models predicting T4 PG-13-R scores. These models will control for study site, time since bereavement, and treatment arm, and they will include either T2 preparedness for patient death or the ACP Execution Checklist score as the focal predictor.

### Interim analyses {21b}

Not applicable.

### Methods for additional analyses (e.g. subgroup analyses) {20b}

All detailed analyses comparing study arms (MCP-C versus SP-C) will be adjusted for randomization stratification variable (study site).

### Methods in analysis to handle protocol non-adherence and any statistical methods to handle missing data {20c}

Efforts to minimize missing data include ensuring an efficient and organized trial design, robust training of research staff, and reducing participant burden by utilizing remote study assessments rather than inperson visits. For participants who report time constraints or difficulty completing study assessments, we will ask them to only complete primary outcome measures (LAP-R and FACIT SWBS) in order to minimize missing data regarding primary study aims. Missing data will be treated using sensitivity analyses and other data analytic approaches that are responsive to missingness. Specifically, we will first exam whether data missingness is associated with variables such as randomization arm and baseline outcome measures by comparing caregivers with complete data to those with missing data. If data is not missing at random, then we will modify analyses by controlling for factors associated with data missingness.^60,61^

### Plans to give access to the full protocol, participant level-data and statistical code {31c}

Reasonable requests for the study protocol or de-identified data can be made to the Principal Investigator.

## Oversight and monitoring

### Composition of the coordinating centre and trial steering committee {5d}

The study team at the coordinating center, ISMMS, is comprised of the CRC and study Principal Investigator. Co-Investigators are housed at MSK and SCCC. The study team at the coordinating site meets weekly to discuss recruitment, active participant, and study questionnaire completion updates. There is no trial steering committee for the current study.

### Composition of the data monitoring committee, its role and reporting structure {21a}

ISMMS/Tisch Cancer Institute (TCI) is serving as the IRB of record, and as such, the data and safety monitoring plan is standard to clinical trials completed at TCI. The TCI Data and Safety Monitoring (DSM) Plan ensures that all clinical trials are high quality, routinely monitored, and meet sponsor, institutional, and governmental requirements. The Plan includes all required elements of the DSM as defined by the NIH and the NCI: monitoring responsibilities, description of what is to be monitored (e.g., data types), frequency of reviews, protocol compliance auditing procedures, adverse event reporting procedures, suspensions of clinical research, and conflicts of interest.

A formal Data and Safety Monitoring Committee (DSMC), independent of the sponsor, was established in 2008 to ensure that all clinical trials that are greater than minimal risk at TCI have adequate data and safety monitoring plans. Interventional, Institutional studies, Investigator Initiated Trials (IIT) requesting that the TCI DSMC serve as the independent Data and Safety Monitoring Board – as is the case with the current investigation – undergo review of the protocol and Data and Safety Monitoring Charter prior to review by the Protocol Review and Monitoring Committee (PRMC) review. The PRMC is charged with the review of all new cancer-related clinical research protocols at Mount Sinai to assess their scientific merit and prioritize them relative to the mission and scope of the TCI. The PRMC coordinator submits the protocol and charter to the DSMC for pre-review. The DSMC Chair, in coordination with the DSMC Administrator, assigns each initial review to a physician and a biostatistician based on the member’s disease specialty and accounting for conflicts of interest. The protocol is submitted before a monthly DSMC meeting, and questions for the PI and responses are documented in minutes. Protocol review includes a plan of review for each arm, dose level or stratum including the number of participants, predefined toxicities, dose adjustments, reporting structures for unanticipated problems and serious adverse event, response assessments, timing of reviews, dose limiting toxicities, dose escalation rules, stopping criteria of the trial for toxicity or efficacy, and criteria defining maximum tolerated dose. Approval of the monitoring plan and charter is required before PRMC approval and is reviewed annually.

Further details are available in the TCI DSMC Charter.

### Adverse event reporting and harms {22}

This is a minimal risk study, and we will only report Serious Adverse Events (SAEs) including deaths that are believed to be at least possibly related to the study intervention or participation. An adverse event is considered any of the following: death, a life-threatening adverse event, an adverse event that results in inpatient hospitalization or prolongation of an existing hospitalization, a persistent or significant incapacity or disruption to the ability to conduct normal life functions, a congenital anomaly/birth defect, and other important medical events that may not result in the above events but may jeopardize the patient or participant based on medical judgment and may require medical or surgical intervention to prevent one of the outcomes mentioned above.

### Frequency and plans for auditing trial conduct {23}

Quality assurance reports will be generated by the study team throughout the study period to monitor participant accrual and completeness of participant registration data. Data quality reports will also be generated to assess data missingness or inconsistencies. Data from the first five participants will be additionally audited for data and consent/eligibility completeness, accuracy, and psychological symptom levels. Thereafter, random samples of data will be assessed for quality and protocol compliance.

### Plans for communicating important protocol amendments to relevant parties (e.g. trial participants, ethical committees) {25}

All protocol amendments are reviewed by study investigators and approved by the appropriate Institutional Review Board ethics committees at each study site.

### Dissemination plans {31a}

Data from the current trial will be disseminated through peer-reviewed journal articles.

## Discussion

This trial builds on a previous pilot trial of MCP-C in caregivers of patients with glioblastoma multiforme that found preliminary evidence of improvements in meaning and purpose, spiritual well-being, and benefit finding. Here, this study protocol describes a fully powered randomized controlled trial to more comprehensively establish the efficacy of MCP-C versus SP-C on enhancing meaning, spiritual wellbeing, anxiety, depression, benefit finding, and social support, as well as on pre- and post-loss bereavement-related outcomes. Findings from this study will contribute to knowledge on the effects of MCP-C on existential distress, spirituality, and other psychosocial outcomes. It will also extend the psychosocial oncology and caregiving science literature to better understand the impact of MCP-C on bereavement outcomes in a diverse sample of caregivers of patients with advanced, solid-tumor cancers.

### Trial status

This trial (Protocol Version 5) is currently recruiting and enrolling caregivers, collecting data, delivering the interventions, and collecting follow-up data. Recruitment began on 3/13/24 and the first participant was consented on that same day. We anticipate completing recruitment by March, 2028.

## Data Availability

Data and materials are available upon reasonable request and agreement from the study PI. The Study PI and biostatistician will have access to the final trial dataset.

## Abbreviations

ACP: Advance Care Planning
BFS: Benefit Finding Scale
CRC: Clinical Research Coordinator
FSSQ: Duke-UNC Functional Social Support Questionnaire
HADS: Hospital Anxiety and Depression Scale
ISMMS: Icahn School of Medicine at Mount Sinai
LAP-R PMI: Life Attitude Profile-Revised, Personal Meaning Index
MCP-C: Meaning Centered Psychotherapy for Cancer Caregivers
MSK: Memorial Sloan Kettering Cancer Center
SAE: Serious Adverse Events
SCCC: Sylvester Comprehensive Cancer Center
SP-C: Supportive Psychotherapy for Caregivers
SWBS: FACIT Spiritual Well-Being Scale
